# Disturbance of a rare seabird by ship-based tourism in a marine protected area

**DOI:** 10.1371/journal.pone.0176176

**Published:** 2017-05-10

**Authors:** Timothy K. Marcella, Scott M. Gende, Daniel D. Roby, Arthur Allignol

**Affiliations:** 1 Oregon Cooperative Fish and Wildlife Research Unit, Department of Fisheries and Wildlife, Oregon State University, Corvallis, Oregon, United States of America; 2 National Park Service, Glacier Bay Field Station, Juneau, Alaska, United States of America; 3 U.S. Geological Survey-Oregon Cooperative Fish and Wildlife Research Unit, Oregon State University, Corvallis, Oregon, United States of America; 4 Ulm University, Institute of Statistics, Ulm, Germany; U.S. Geological Survey, UNITED STATES

## Abstract

Managers of marine protected areas (MPAs) must often seek ways to allow for visitation while minimizing impacts to the resources they are intended to protect. Using shipboard observers, we quantified the “zone of disturbance” for Kittlitz’s and marbled murrelets (*Brachyramphus brevirostris* and *B*. *marmoratus*) exposed to large cruise ships traveling through Glacier Bay National Park, one of the largest MPAs in North America. In the upper reaches of Glacier Bay, where Kittlitz’s murrelets predominated, binary logistic regression models predicted that 61% of all murrelets within 850 m perpendicular distance of a cruise ship were disturbed (defined as flushing or diving), whereas in the lower reaches, where marbled murrelets predominated, this percentage increased to 72%. Using survival analysis, murrelets in both reaches were found to react at greater distances when ships approached indirectly, presumably because of the ship’s larger profile, suggesting murrelets responded to visual rather than audio cues. No management-relevant covariates (e.g., ship velocity, route distance from shore) were found to be important predictors of disturbance, as distance from ship to murrelet accounted for > 90% of the explained variation in murrelet response. Utilizing previously published murrelet density estimates from Glacier Bay, and applying an average empirical disturbance probability (68%) out to 850 m from a cruise ship’s typical route, we estimated that a minimum of 9.8–19.6% of all murrelets in Glacier Bay are disturbed per ship entry. Whether these disturbance levels are inconsistent with Park management objectives, which include conserving wildlife as well as providing opportunities for visitation, depends in large part on whether disturbance events caused by cruise ships have impacts on murrelet fitness, which remains uncertain.

## Introduction

A recurring issue in the management of marine protected areas (MPAs), particularly those focused on biodiversity conservation, is finding optimal solutions to conflicting mandates. For example, MPAs that function to conserve habitat utilized by upper trophic level organisms, such as marine mammals and seabirds, are regularly targeted for ‘non-consumptive’ ecotourism activities [1–3]. These non-extractive activities, such as whale-watching, are often promoted because they can provide direct economic benefits to gateway communities, thereby increasing the local acceptance of the MPA and ultimately enhancing chances for success [4–6]. Ecotourism activities in MPAs are also assumed to have lower impacts on population persistence of upper trophic species compared to consumptive activities, such as commercial fishing, which may result in incidental mortalities due to by-catch [7].

Despite its non-consumptive nature, ecotourism activities can negatively affect upper trophic level organisms, rendering management decisions regarding sustainable use and levels of visitation difficult. For example, many ecotourism activities result in behavioral disturbance to a subset of individual wildlife. While behavioral changes may have little energetic, physiological, or fitness-related impacts (e.g., [8]), recent studies have demonstrated clear linkages between behavioral changes and reduced vital activities of marine wildlife [9–11]. In some cases, seemingly benign transitions of behavioral states may ultimately have population-level consequences [12] that may not be detected for years [13]. Critical to making informed decisions about appropriate levels and types of ecotourism, then, is a robust accounting of impacts on wildlife behavior, even if these effects cannot be directly linked to an immediate estimate of population-level consequences.

Glacier Bay National Park and Preserve (Glacier Bay or “the Park”) provides an excellent case study for the types of tradeoffs commonly faced by MPA managers. Located in southeastern Alaska, Glacier Bay is part of the 24.3 million-acre Kluane/Wrangell-St. Elias/Glacier Bay/Tatshenshini-Alsek World Heritage Site, which includes over 600,000 acres of marine ecosystems making it one of the largest marine protected areas in the United States. Glacier Bay is a coveted destination for visitors to Alaska because it includes pristine coastal forests, a number of tidewater glaciers, productive marine ecosystems [14], and a diverse array of marine wildlife, including large aggregations of marine mammals and seabirds [15–17]. Like other National Parks in the U.S., management of Glacier Bay has a dual mandate of both resource conservation and providing for “…appropriate opportunities to experience enjoyment of the [Park’s] resources” (see p.11 in [18]). Owing to a lack of roads connecting the Park to outside areas of Alaska, nearly all visitors access the Park via marine vessels [19]. Therefore, a recurring management issue revolves around making informed decisions regarding opportunities for visitors to experience the Park while still conserving “…the natural abundances…and behaviors of native plant and animal populations” (see p.48 in [18]).

Here we examine the issue of disturbance by visitors to a seabird of considerable conservation concern, the Kittlitz’s murrelet (*Brachyramphus brevirostris*) [20–21], a small pursuit-diving seabird in the family Alcidae endemic to Alaska and the Russian Far East [22]. While there is significant uncertainty regarding its range-wide status [23–25], the Kittlitz’s murrelet is a priority conservation concern for the National Park Service (NPS) because up to 37% of the world’s known population of the species may utilize the Park during the spring and summer months [26]. What’s more, large vessels are known to regularly disturb murrelets, with potential consequences to their foraging and nesting activities [27]. Disturbance of murrelets by visitors also generates a conflict with the goal of preserving natural behaviors, and potentially with the goal of population viability [18].

We focused our study on large cruise ships because these vessels play a disproportionately important role in supporting visitation to the Park compared to other vessel types. Since 2007, nearly 225 cruise ships have entered the Park each year carrying >400,000 passengers, or typically > 95% of all annual visitations to the Park [19]. Therefore, any management decision intended to reduce disturbance to murrelets (or other wildlife) by cruise ships would simultaneously affect the overwhelming majority of visitors to the Park. Also, unlike the management of other marine vessels, the Park Superintendent must make an annual determination regarding the number of allowable cruise ships within the Park, requiring a robust evaluation of the impacts of such a management decision.

Our overarching goal was to identify the extent to which cruise ships disturb murrelets by quantifying the ‘zone of disturbance’ created when they travel on their regular route through Glacier Bay. To this end, our fundamental objective was to identify the change in probability of disturbance as a function of the perpendicular distance from the ship’s course, including any biological, environmental, or management-relevant covariates that might influence the width of this zone. We also sought a better mechanistic understanding of events resulting in disturbance to murrelets; consequently, we used survival analysis to model variation in murrelet disturbance response relative to dynamic approach angles by cruise ships. Using these disturbance probabilities and long-term monitoring data [28], we then generated a minimum estimate of the total number of murrelets that are disturbed by a cruise ship traversing Glacier Bay. While we cannot infer population consequences from this study, we sought to identify any factors regulated by the NPS, such as ship speed or number of ships in the Park, that may influence the size or extent of the zone of disturbance providing managers with options should disturbance rates be deemed inconsistent with Park objectives.

## Methods

### Study area

**Fig 1:**
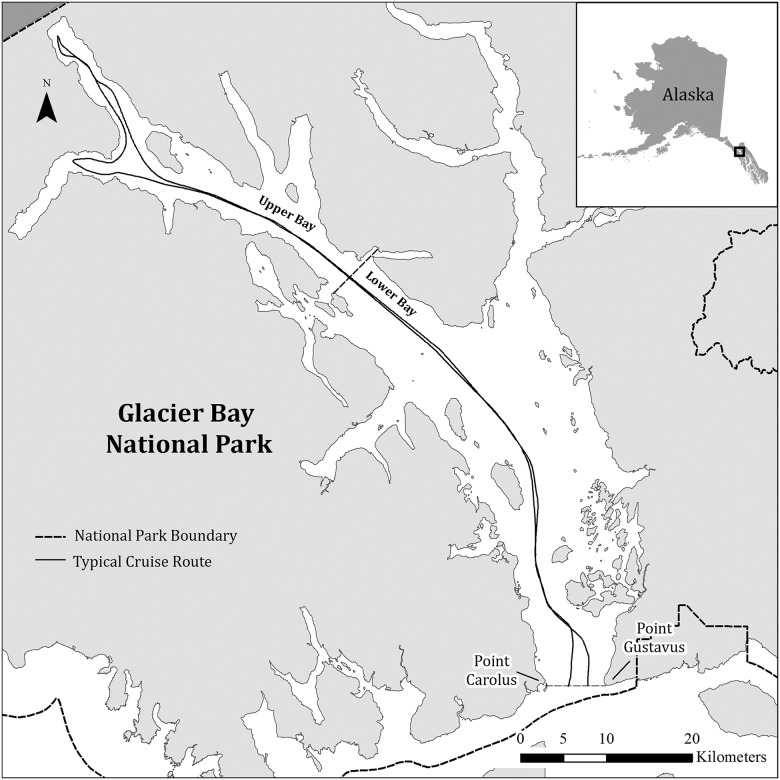
Map of Glacier Bay in Glacier Bay National Park, Alaska. Gray dashed line indicates the Park boundary. Solid gray line indicates a typical cruise ship route within the Bay. The black bar indicates the dividing line between the Upper Bay and the Lower Bay sectors of the study area.

Glacier Bay National Park and Preserve is located in southeastern Alaska, 100 km northwest of Juneau. Although the Park includes areas on the outer coast adjacent to the open North Pacific Ocean, much of the viewable wildlife and all the tidewater glaciers, and thus the focus of Park visitation, occurs in the protected, 110-km, Y-shaped fjord, commonly referred to as Glacier Bay (Fig 1; 58.761 Latitude, -136.3483 Longitude). The Park’s jurisdiction includes both the land and marine waters, making it one of the few “ocean parks” in the U.S. National Park System.

### Cruise ships in Glacier Bay

The NPS regulates the number and type of marine vessels allowed in Glacier Bay by designating daily and seasonal quotas [29]. For cruise ships, the maximum daily quota is two entries per day and does not vary from year to year. The seasonal quota, however, is decided annually, and split into a 92-day peak season (1 June– 31 August) and a 61-day shoulder season (May, September). The peak season quota is currently set at 153 cruise ship entries. Most days during the peak season, the daily maximum of two cruise ships visit the Park, but there are still a number of days when one or no cruise ships are permitted to enter the Park. The Park Superintendent may reduce this quota based on resource impact concerns, or may increase the peak seasonal quota up to its maximum of 184 (two ships every day for 92 days) [29]. Currently the shoulder seasonal quota for cruise ship entries is set at 92, although weather conditions and other factors limit the market demand for Alaska cruises during this period.

For other vessels, the peak season daily quota includes 3 tour vessels, 2 charter vessels, and 25 private vessels. In both 2011 and 2012, the average number of private vessels present in Glacier Bay was 18 and ranged between 6 and 25 (NPS, unpublished data). The majority of the private vessel boat activity occurs in the lower parts of the Bay and is centered around Bartlett Cove where NPS administrative offices are located.

The operations of cruise ships that enter Glacier Bay are remarkably similar. Cruise ships enter the Park at the mouth of Glacier Bay in the morning, generally between 06:00 and 10:30 ADT, and proceed up the fjord until reaching the tidewater glaciers at the head of the West Arm of the Bay three to four hours later (Fig 1). During their transit, cruise ships are required to remain mid-channel and, due to navigational hazards, tend to follow the same general route [30] (Fig 1). Ships typically spend four to five hours in the upper West Arm before proceeding back along the same route, exiting 9–11 hours after entering. Most variation in visitation time can be attributed to speed restrictions that may be in place in certain parts of the Park for humpback whale (*Megaptera novaeangliae*) conservation measures [29], or from time constraints owing to the need to make the next port of call. No large cruise ships are present in the Park overnight, and all passengers remain on board the ship during the entire visit.

We note that cruise ships provide some inferential advantages to understanding the magnitude of disturbance to wildlife: in addition to taking nearly identical routes during each visit, they also cannot abruptly change course or speed, which could confound observational efforts [31]. Cruise ships in the Park also do not change their operations due to the presence of the observer on deck.

### Experimental design

All field work was conducted with permission from the National Park Service, Glacier Bay National Park under permit #GLBA-2011-SCI-0001. Prior to conducting murrelet observations from cruise ships, observers spent several weeks training in Glacier Bay to accurately identify murrelets to species at a distance (Kittlitz’s or marbled murrelet [*B*. *marmoratus*]). Beginning in June and continuing through mid-August in both 2011 and 2012, murrelet behavior was recorded by observers while onboard a total of 13 different cruise ships from either the Holland American Line (HAL) or Princess Cruise Lines (Princess) during 45 cruises into Glacier Bay (N = 22 cruises in 2011 and N = 23 cruises in 2012). Observation time onboard the ships averaged just over six hours per cruise in each year of the study (range = 3.5–8.0 h). Cruise ship sizes were typical of those operating in Alaska and in Glacier Bay, ranging in length from 228 to 294 m and 29 to 37 m wide at the beam. HAL and Princess ships account for over 60% of the cruises into Glacier Bay, and thus our results are representative of the population of cruise ships that enter the Park annually.

For our study, we built on successful methods developed by Jansen et al. [32] and Harris et al. [33], wherein an observer stood at the forward-most position on the bow of a cruise ship with rangefinder binoculars and, with unencumbered views of the waters in front and adjacent to the ship, recorded responses of murrelets to the ship as it traveled within the Park. Observations were made using a pair of rangefinder binoculars (Leica Vector IV, 7x 42mm, Heerbrugg, Switzerland; range up to 6 km +/- 1 m) mounted on a tripod (Manfrotto 3021BPRO, Upper Saddle River, NJ, USA) with an attached compass rose (compass rose only used in 2012). For estimating disturbance, our protocol dictated that observers haphazardly chose either a single murrelet, or one murrelet in a group of murrelets (focal murrelet), as far from the ship as possible, and at varying bearings off the ship’s heading. Groups of murrelets were defined as two or more birds within ~two meters of each other or multiple murrelets acting in unison (e.g., swimming in a line). While large ‘aggregations’ of murrelets were encountered on occasion, 86% of the 4,251 observations used in our analysis were of group sizes of 3 or less, 93% were of group sizes of 5 or less, and the largest group size we encountered was 15 individuals. We note that our definition of a ‘group’ reflected that of Hoekman et al. [34], who defined a group as an association of individuals separated by < ~5 m. Consequently, the average group size found during our 2-year study ($\bar{x}$ group size = 2.07; SD = 1.13) was similar to the average group size across those same years found during independent survey estimates (2011: $\bar{x}$ group size = 1.68; SD = 1.09; 2012: $\bar{x}$ group size = 2.11; SD = 1.52; [34]). Thus, we feel our definition of group size accurately distinguished individuals associated with each other vs. a larger aggregation.

Observations were initiated only on “undisturbed” murrelets on the surface of the water, which we termed “loafing”. We defined loafing as including any behavior other than flying or diving, and encompassed a range of behaviors conducted on the surface of the water including comfort (resting/sleeping), maintenance (preening), or vigilance (alert, calling, swimming away). We recognize that by defining vigilant murrelets as undisturbed we are underestimating the true rate of disturbance. However, owing to the much larger energetic consequences of flight and dive responses compared to vigilance and swimming from the ship, plus difficulties in determining when vigilance or swimming from the ship by murrelets first occurred, we chose to define taking flight (flushing) as the primary response to disturbance and diving as the secondary response.

In addition to the distance of the observer from the focal murrelet, we also recorded the location of the bird relative to the cruise ship’s heading (the relative bearing which we define as the bearing). Because the values of both distance and bearing change as the ship approaches the focal murrelet (i.e. are distance-dependent), repeated measurements were collected approximately every 10 sec until the focal murrelet reacted by flushing or diving, or the observation was terminated when the murrelet passed abeam of the ship’s bow. Additionally, for each focal murrelet we also recorded: (1) species of murrelet, if discernable, (2) murrelet group size, (3) Beaufort wind speed, (4) whether there were one to two cruise ships in the Park that day, and (5) number of days since June 1 (as a measure of seasonality). Ship location and velocity data were collected using a handheld Garmin GPS (GPSMAP 76Cx, Olathe, KS, USA) set to record a location every five seconds during the cruise. Velocity, location, and distance to shore were considered ‘management relevant’, i.e. variables that could be regulated to reduce disturbance to murrelets by ships if those variables were found to significantly explain variation in flushing probability. Distance to shore and location are important variables explaining differences in the distribution of murrelets [34]. Thus, if flushing probability is related to either of these variables, the Park could alter the routes used by ships to minimize disturbance.

Ship speed was calculated as a ratio of the distance covered per 60-sec period centered on the observation time, and was converted to nautical miles per hour (knots; see also [35]), whereas data on ship distance from shore and location within the Park were generated using the GPS data and basic tools in ArcMAP 10.0 [36]. Although these variables could have changed slightly over the course of one focal murrelet observation, they were considered fixed for all repeated measurements of a particular focal murrelet. Observational data were dictated in real time into a hands-free digital voice recorder (Olympus DS2400, Centerville, PA, USA). The recorded data were later played back using Wave Pad Sound Editor v 4.52 [37] and entered into a digital database.

The forward-most point on a cruise ship from which observations were made resulted in the observer being an average of 15.2 m (range: 14.3–15.5 m) above the water. Thus, the distance to a focal murrelet recorded from this height differed slightly from the distance at waterline. We chose not to correct for this discrepancy as murrelets are likely reacting to the entire ship, not just the portion at the waterline. We nevertheless only make statements about reaction probability at a coarse scale (50 m increments). The configuration of the bow prevented observers from viewing murrelets that were closer than about 50 m directly in front of the ship or closer than about 100 m abeam, although our results demonstrate that nearly all focal murrelets reacted before being approached at such close distances. The area surveyed by the observer included the water surface 1,000 m to the front and side of the bow of the cruise ship, and alternated between port and starboard sides of the cruise ship during consecutive cruises. Observations were collected only while the ship was traveling through the Bay, and were temporarily terminated when the ship was stopped in front of tidewater glaciers or when fog or heavy rain impaired visibility.

Owing to the small size of murrelets, the height of observers above the water, and the similarity in plumage and profile between Kittlitz’s and marbled murrelets, we encountered two primary sources of observational error that could have introduced bias into our results. First, the rangefinder binoculars were generally unsuccessful in measuring murrelets at distances of greater than 500 m, requiring distances from 500 m to 1,000 m to be visually estimated. To examine this potential bias in estimating distances, we located objects in the water throughout the field season (e.g., icebergs, logs, or birds) that could be targeted using the rangefinders and visually estimated their distances immediately before measuring the distance with the rangefinder binoculars (2011; N = 417). In 2012, rather than separate objects, we similarly estimated the distances to every focal murrelet immediately before recording the distance with the rangefinders (2012; N = 917). We then subtracted the measured value from the corresponding estimated value and calculated the mean average error (AVEerror) and the root mean square error (RMSE) for both 2011 (AVEerror = 48.5 m, RMSE = 68.0 m) and 2012 (AVEerror = 47.6 m, RMSE = 62.0 m). Empirical cumulative distribution functions of the errors in each year were then overlaid onto normal distribution functions (See S1 Appendix). A two-sample Kolmogrov-Smirnov test confirmed the distribution in errors between the two years were not significantly different (D = 0.034, *P* = 0.887; [38]). Because no systematic bias appeared to be present when distances were estimated rather than measured, either within or between years, we chose not to treat estimated distances differently from measured distances in the statistical analysis.

Our second major source of observer error, and potential source of bias, was identification of murrelet species. Owing to their unique body size and profile among birds found within the study area, Kittlitz’s and marbled murrelets were easily differentiated from other species of waterbirds at distances up to 1,000 m. However, distinguishing between the two murrelet species required detecting slight differences in plumage characteristics and bill size [22] that are increasingly difficult to discern at distances in excess of 300 m. Perhaps more importantly, our ability to distinguish between the two species differed depending on their response to the cruise ship—murrelets that flushed from the water allowed much better detection of interspecific plumage differences and a longer time to observe the focal bird compared to those that dove. At distances between 300 m and 400 m, we identified to species 65% of murrelets that flushed from the water, but only 11–13% of murrelets that dove or remained on the water’s surface. Focal murrelets that responded to the approaching ship at distances > 400 m were usually not identified to species, regardless of response.

**Fig 2:**
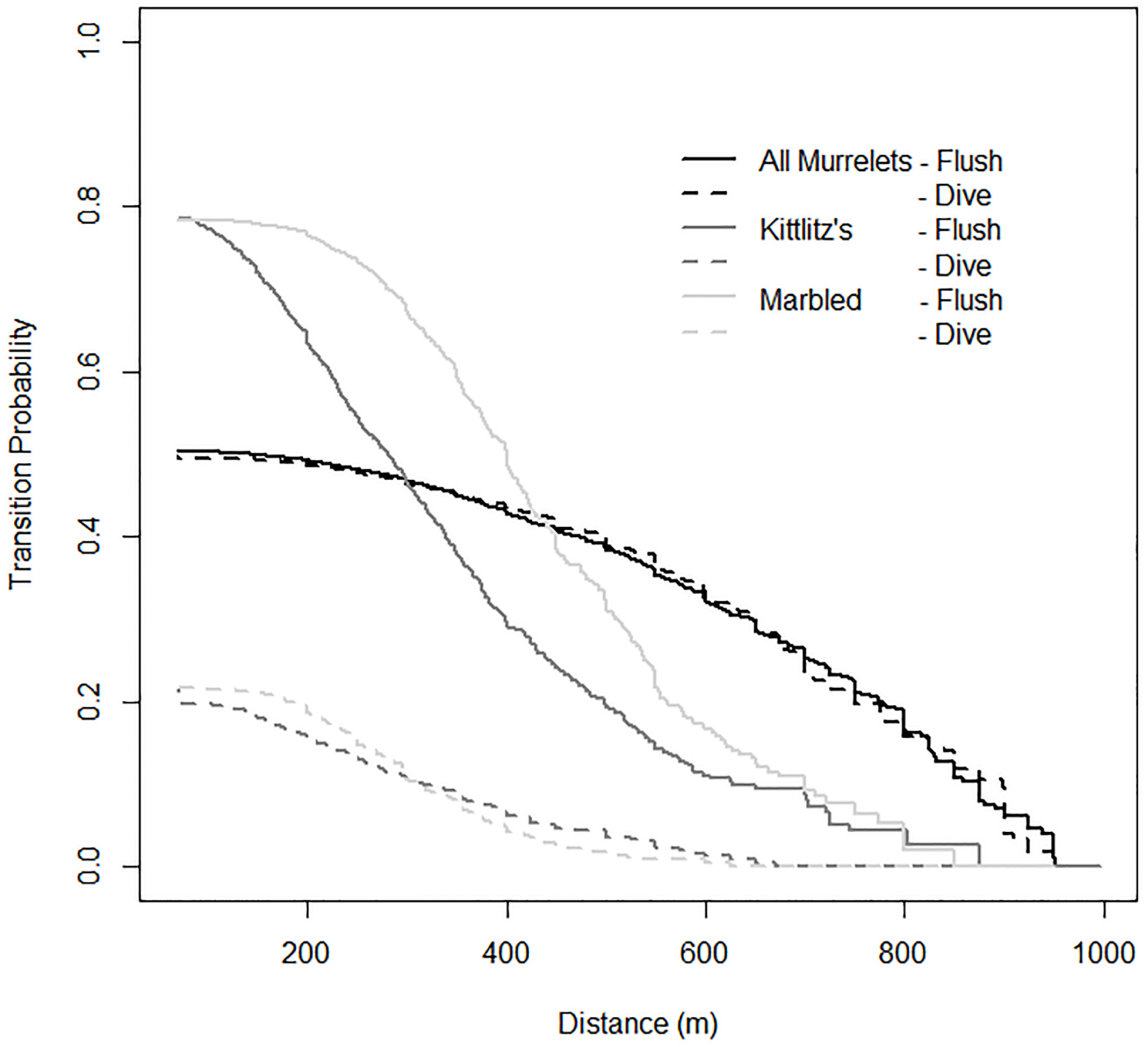
Cause-specific cumulative incidence curves indicating the transition probability from loafing to flying or diving for murrelets (all murrelets, Kittlitz’s murrelets, and marbled murrelets) as a function of approach distance by cruise ships in Glacier Bay National Park, Alaska, during 2011 and 2012.

To examine the magnitude of bias in species identification relative to murrelet response, we used the ‘etm’ package in R [39] to calculate cause-specific estimates of the cumulative incidence function for the probability of flushing and diving for (1) identified Kittlitz’s and marbled murrelets separately, and (2) all murrelet observations (identified + unidentified) combined. The cumulative incidence function at a given distance is the probability of experiencing a particular event (flushing or diving) prior to that distance and prior to experiencing any other competing events. Plotting the cumulative incidence functions for all murrelets (identified and unidentified) demonstrated a strong bias towards greater identification rates for murrelets that reacted by flushing as opposed to diving, but an equal occurrence of flushing and diving reactions across the range of sampled distances from the ship for all murrelets (Fig 2).

In order to avoid estimates of species-specific disturbance distances that were biased low, we chose to lump all observations and conduct all further analyses at the genus level. However, 79% of identified murrelets in the Upper Bay were Kittlitz’s murrelets and 83% of identified murrelets in the Lower Bay were marbled murrelets, suggesting habitat partitioning by the two species during the study period (see also [28]). In an attempt to explore species-specific response differences, we generated a dichotomous variable for our models (Upper Bay vs. Lower Bay) representing habitat partitioning during the two years of our study as a covariate in the genus-level analysis. Using this approach, we lose the ability to make definitive statements about inter-specific differences in both reaction distance and reaction type, but preserve the ability to accurately assess and compare the two types of behavioral response (flush vs. dive) of murrelets encountered by cruise ships in Glacier Bay.

### Probability of disturbance

#### Logistic regression

From a management perspective, the NPS seeks to understand the zone of disturbance as a result of allowing cruise ships to enter the Park, i.e. how far on either side of the ship’s course are murrelets likely to be disturbed and, by extension, which areas of the Park constitute habitat free of cruise ship disturbances. To this end, we calculated the perpendicular distance to the cruise ship’s course using planar geometry from the last collected distance and bearing angle for each focal murrelet. Murrelets responding to the approaching ship (either by flushing or diving) prior to passing abeam of the bow were scored as 1, while those remaining on the water as the ship’s bow passed abeam were scored as 0. Observations that were terminated prior to the focal bird reacting or passing abeam were eliminated from this analysis. Binary logistic regression was used to estimate the probability of a murrelet responding to the ship prior to passing abeam using distance as a continuous explanatory variable, among other biological and management-relevant covariates (see Table 1 for covariates explored). The best fit model was chosen by stepwise AIC model selection using the ‘MASS’ package in R [40]. For ease of consideration for management purposes, interaction terms were not investigated as part of this analysis. Model fit was assessed by investigating the receiver operator characteristic (ROC) and calculating the area under the curve (AUC) statistic using the ‘ROCR’ package in R [41].

**Table 1:** Explanatory variables considered when modeling the response of murrelets to approaching cruise ships in Glacier Bay.

Variable (sample size)	Classification
**Year**	
2011 (N = 2,365)	Year observation was collected
2012 (N = 1,886)	
**Location in Park**	
Upper Bay (N = 2,040)	North of Tidal Inlet to head of the Bay
Lower Bay (N = 2,211)	South of Tidal Inlet to Ripple Cove in lower Bay
**Group Size**	
Single (N = 1,181)	Solitary murrelet
Group (N = 3070)	≥ 2 murrelets within 2 meters or acting in concert
**Ship Entries Per Day**	
One (N = 1,517)	Only one cruise ship in the Bay during the day
Two (N = 2,734)	Two cruise ships in the Bay during the day
**Sea State**	
Light (N = 3,701)	0 or 1 on the Beaufort Sea State Scale
Moderate (N = 550)	2 or greater on the Beaufort Sea State Scale
**Ship Speed**	
continuous	Nautical miles per hour (knots)
**Distance to Shore**	
continuous	Kilometers from nearest shoreline
**Stage of Season**	
continuous	Days since June 1

Sample sizes included in parentheses.

Given that a primary purpose of this study was to provide managers with an average reaction distance for Kittlitz’s murrelets in response to cruise ships traveling through Glacier Bay, we chose to not include year as an explanatory variable in models explored, allowing the covariate effects to reflect the average murrelet reaction across both study years.

#### Survival analysis—Multistate modeling approach

Using only the last data point (disturbed or not) and only the perpendicular distance from the ship’s course fails to utilize information about responses by murrelets that may lead to better management or insight into biological or environmental factors that influence responses, such as approach angles. To further investigate mechanistic factors that influence murrelet disturbance response (flush or dive) across a range of approach angles, as well as environmental and management-related variables, we used survival analysis.

Survival analysis, also referred to as event time analysis, is commonly used for analyzing the amount of time that elapses before an event of interest occurs [42]. Typical survival analysis studies originate at time zero (study onset) and follow an individual until the time the event occurs or until the predetermined end of the study. Survival analysis studies often need to account for alternative events that preclude or prevent observation of the event of interest. These situations are referred to as competing risks. Observations may also be terminated prior to experiencing an event of interest when a subject is lost from the study due to an independent or non-informative reason, or remain event-free for the duration of the observation period. Additionally, a subject may enter the study at a time later than the study initiation time. These last two scenarios are referred to as right-censoring and left-truncation, respectively. Methods have been developed to handle these scenarios when maximizing the likelihood of the survival function and the related hazard function [43–44].

Unlike typical survival analysis studies that focus on time-to-event [42], we were interested in the distance-to-event [45–46]. For our study, we were particularly interested in the flushing response because taking off from the water is arguably the most energetically costly avoidance behavior that a passing cruise ship can elicit, as compared to swimming away from or diving in response to the vessel [47]. Diving, however, was also a common response that precluded us from following the individual any longer, and was thus analyzed alongside flushing as a competing risk. Our data are also characterized as being right-censored, as there were certain situations when an event of interest was not observed during the study period, i.e. when a bird was lost from sight due to glare or because it passed abeam of the ship without diving or flushing. Left-truncation was present when observations of focal murrelets were initiated closer than 1,000 m from the ship, the furthest observation distance considered for this analysis, or when a distance-dependent variable changed.

Standard survival analysis deals with the passage of time, wherein the starting time for observation of each subject (e.g., year 1) is necessarily smaller than the ending time (e.g., year 12). For our analysis, however, the distance at which we initiated observations of a focal murrelet was greater than the ending distance -- the ship was constantly getting closer to the focal murrelet. In order to account for this, the data were transformed by subtracting all observation distances from 1,000 m, resulting in the increasing measurement values needed for the analysis. All reaction distances were back-transformed after analysis and prior to presenting results; therefore, all results reflect the distance (in meters) where each murrelet ultimately responded to the ship.

In order to test for an effect of the distance-dependent variable “approach angle,” we needed to effectively censor and truncate observations every time the angle changed. Approach angle was dichotomized into categories of 0° - 44° or 45° - 90° from the ship’s course. Each time the approach angle category changed for a focal murrelet, the observation was censored and a new left-truncated observation began. The truncation process created two pseudo-individuals, each only contributing follow-up information to the estimated hazard when the covariate value was equal to one specific category. By right censorship and left truncation we ensured that each portion of a focal murrelet observation that spanned the two bearing bins was independent of the other [44].

We dichotomized the approach angle into 2 categories, rather than as a continuous variable, because event analysis measures the time (in our case, distance) to an event of interest. In most applications of event analysis, the variables measured are static and do not change (or are assumed not to change) during the course of the study (sex, status, treatment, etc.). If a variable does change during the study, the observation is censored and a “new” observation is started at the point in time of the change in the variable. Censoring an observation thus essentially creates a new pseudo-individual, allowing the observation prior to the variable transition to be independent of the observation post-transition. As such, if a continuous variable is hypothesized to have an effect on the outcome, the variable is grouped or dichotomized to ensure there are sufficient sample sizes in each category.

We developed a multistate Cox regression model that allowed us to investigate the effects of the covariates across the different approach angles and disturbance responses [48]. The process modeled follows a uni-directional, multistate model without recurrent events. Approach angle was a distance-dependent variable because its value could change as the distance between the ship and the focal murrelet decreased. This fact made it possible for a focal murrelet to start in the 0° - 44° approach angle state and transition to the 45° - 90° state, prior to entering an absorbing state by flushing or diving. The transition between transient states is considered uni-directional and non-recurring, as murrelets could not transition from the 45° - 90° approach angle state to the 0° - 44° state without flushing. This process was assumed to follow a Markov property, i.e. only the present state occupied and distance from the ship, not the process leading to this occupation, would affect the subsequent transition [48].

**Fig 3:**
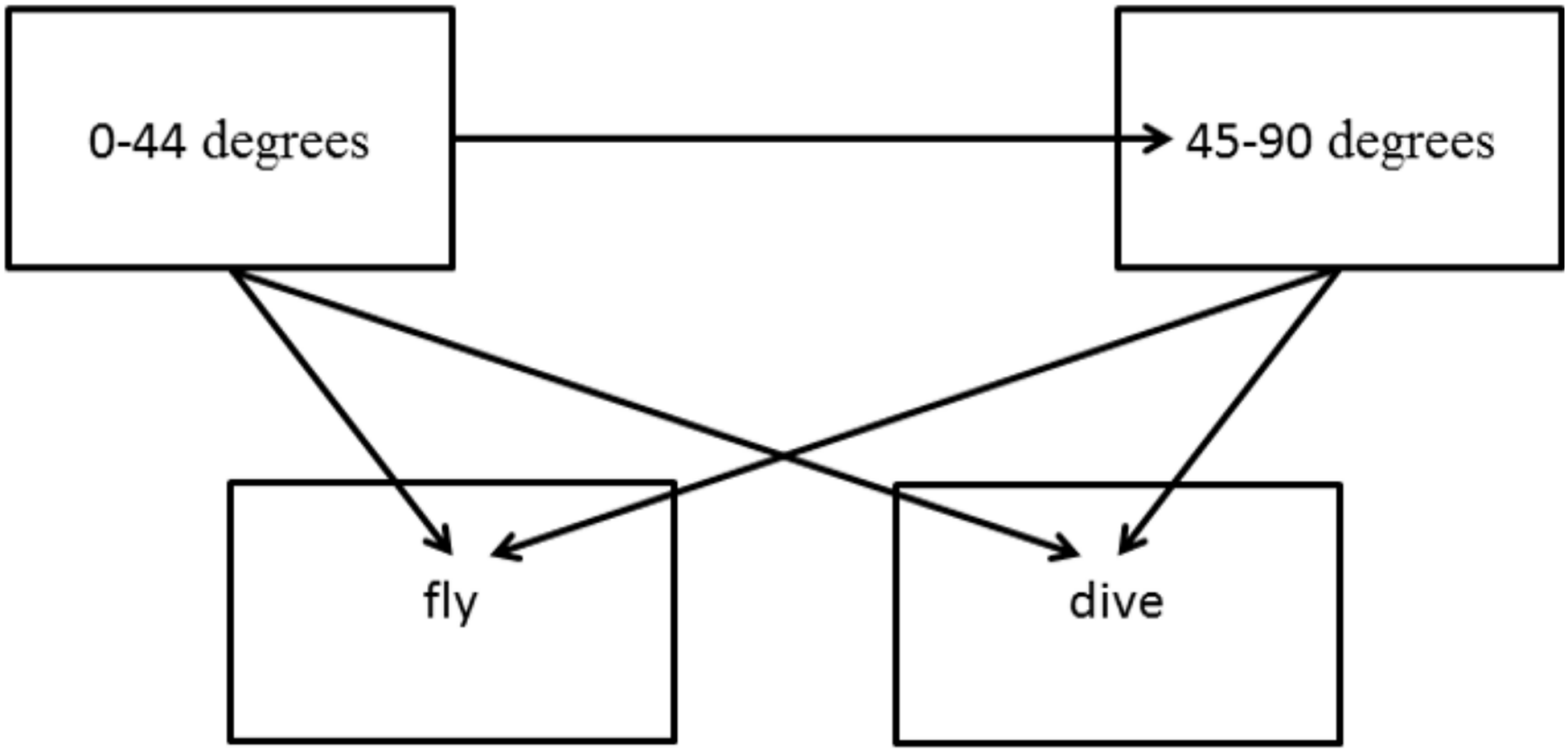
Multistate model depicting two transient states (0° - 44° and 45° - 90° from ship’s course) and the two absorbing states (behavioral responses of flush or dive) for murrelets approached by cruise ships in Glacier Bay, Alaska. Numbers indicate the five possible transitions that were modeled.

The multistate model was constructed using the ‘mstate’ package in program R [49]. Five transitions were considered: one between the transient states (0° - 44° to 45° - 90°), and two each from the transient bearing states to either of two absorbing states (flushing or diving; Fig 3). The effect of each explanatory variable was explored within a non-parametric Cox proportional hazards framework for each of the five transition types [48]. Because sample sizes were large and interpretation of observed behaviors was the goal of the modeling exercise, we chose to include all variables in the final model, even if not statistically significant. The proportional hazard assumption was checked by exploring the Schoenfeld residuals. Interaction terms were not explored in the multistate model.

#### Estimating percent disturbed

Finally, we approximated the total number of murrelets disturbed by a typical cruise ship travelling through Glacier Bay. To do so, we first quantified the total area that fell within the estimated ‘zone of disturbance’ based on our logistic model using a typical cruise ship track [30], and then used the results from an independent study that has generated robust and unbiased estimates of murrelet abundance and density in Glacier Bay during July 2009–2014 [28]. Given that cruise ships essentially utilize the same route moving up Glacier Bay as they do when moving down the Bay, we generated two minimum estimates for the average number of murrelets disturbed per entry assuming that (1) all murrelets disturbed by a cruise ship during its egress from the Bay were independent of individuals disturbed upon the ship’s ingress to the Bay (effectively doubling the total number disturbed), and (2) all those disturbed upon ingress were re-disturbed during egress. As we assumed that cruise ships elicited no disturbance response from murrelets at distances greater than our maximum perpendicular observation distance (850 m), these estimates must be considered minimum estimates of the magnitude of disturbance.

## Results

**Fig 4:**
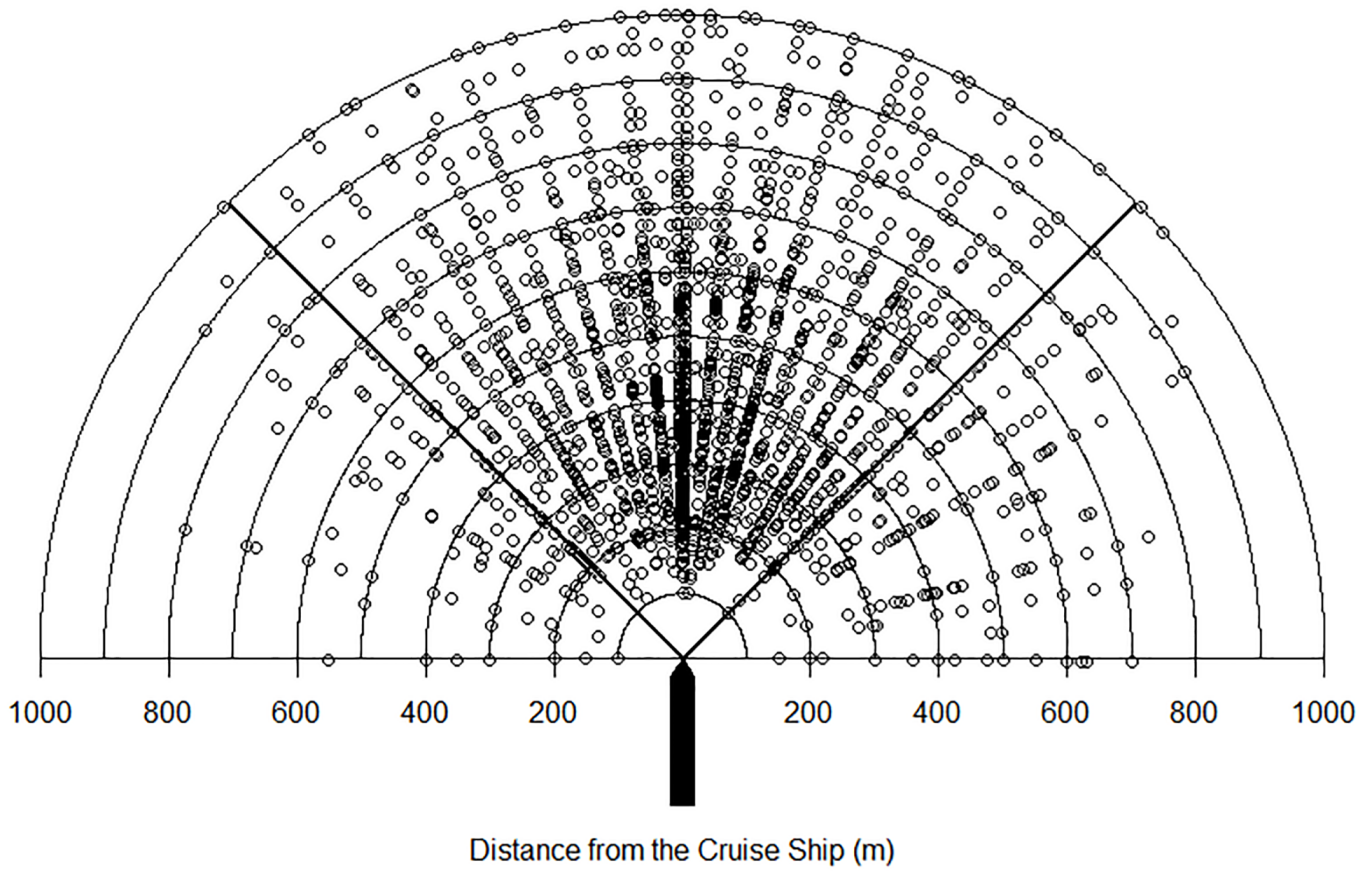
Initial locations of focal murrelets observed from the bow of cruise ships as they traveled through Glacier Bay National Park, Alaska, in 2011 and 2012. The silhouette of the ship is sized to scale and represents the average length of cruise ships that visit Glacier Bay. Wedges to port and starboard of the ship’s course indicate the 45° - 90° bearing bins, while the wedge centered on the ship’s course indicates the 0° - 44° bearing bin.

Over the two years of the study, 4,251 focal *Brachyramphus* murrelets were followed from an initial detection at ≤ 1,000 m from the ship across a range of (relative) bearings until the murrelet flushed, dove, or passed abeam of the ship (Fig 4). A total of 1,191 of the focal murrelets (28.0%) were ultimately identified as Kittlitz’s murrelets, 1,225 (28.8%) were identified as marbled murrelets, and the remaining 1,835 (43.2%) were recorded as unidentified *Brachyramphus* murrelets (Table 2). Most focal murrelets (N = 3,762) reacted to the approaching ship by either flushing or diving, and these two responses occurred with similar frequency (47.0% and 41.5%, respectively). Passing abeam of the ship without flushing or diving accounted for only 5.8% (N = 243) of all observations. Visual contact was lost prior to observing a response (or passing abeam) for the remaining 5.7% of focal follows (Table 2).

**Table 2:** Behavioral response to approaching cruise ships for murrelets identified to species, murrelets unidentified to species, and all *Brachyramphus* murrelets in Glacier Bay National Park, Alaska, during 2011 and 2012. Murrelet species identification rates were negatively affected by both response distance and a dive response compared to a flight response.

Species	Fly	Dive	Pass Abeam	Censored	Total
Kittlitz’s	782 (65.7%)	216 (18.1%)	78 (6.5%)	115 (9.7%)	**1,191**
Marbled	742 (60.6%)	424 (34.6%)	18 (1.5%)	41 (3.3%)	**1,225**
Unidentified	472 (25.7%)	1,126 (61.4%)	147 (8.0%)	90 (4.9%)	**1,835**
**Total**	**1,996 (47.0%)**	**1,766 (41.5%)**	**243 (5.7%)**	**246 (5.8%)**	**4,251**

### Response distance—Logistic models

**Fig 5:**
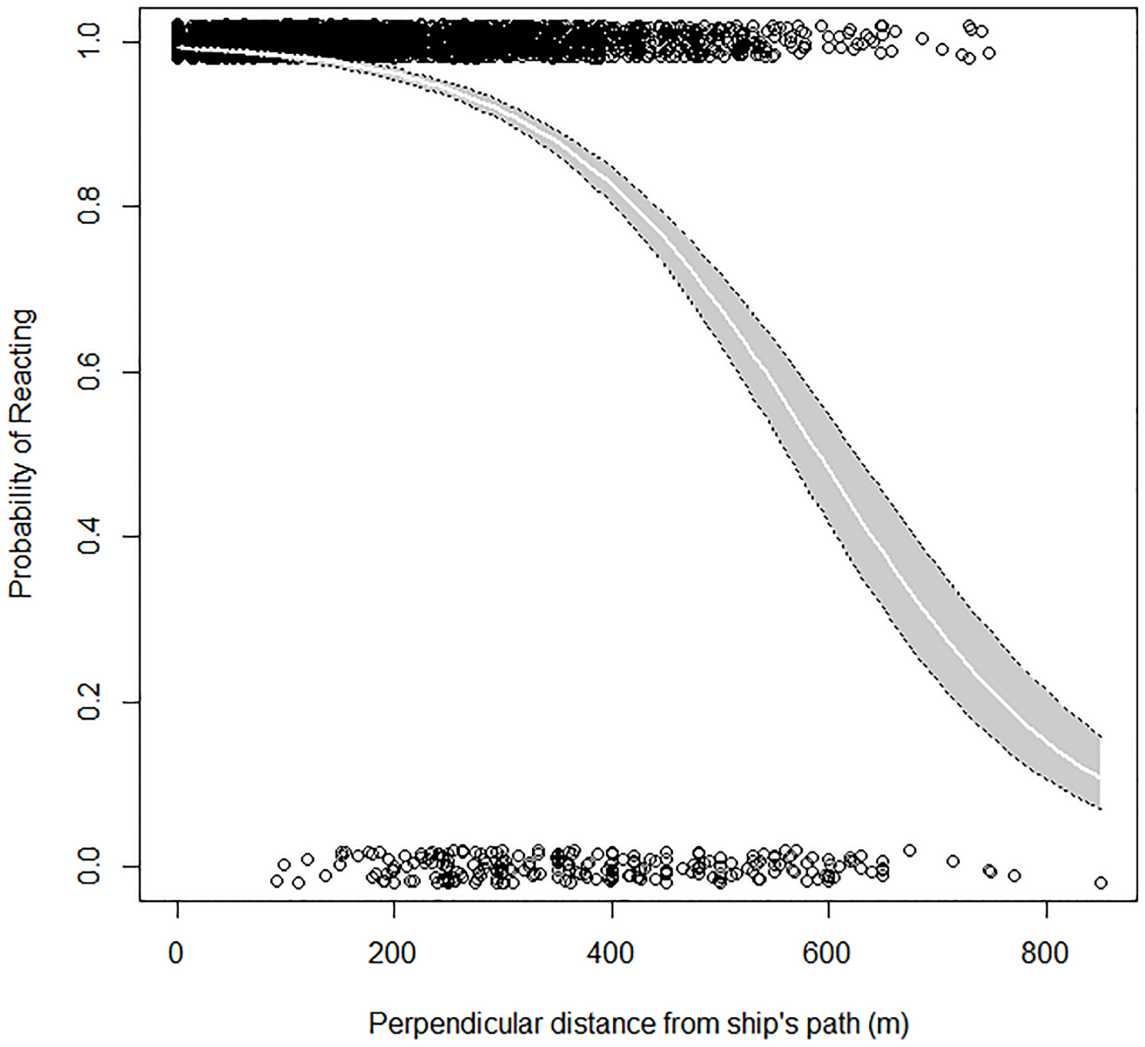
Fitted binary logistic curve representing the observed probability of response (either flushing or diving) by murrelets encountered on the water as a function of the murrelet’s perpendicular distance from the cruise ship’s course (either to port or starboard). Shaded region represents the 95% confidence interval. Points were jittered so as to better reflect the distribution of responses vs. no responses. Data were collected from onboard cruise ships in Glacier Bay National Park, Alaska, during 2011 and 2012.

The perpendicular distance of a murrelet to the cruise ship’s course accounted for approximately 93% of the explained variation in disturbance response; seasonal and location effects accounted for a significant but small amount of the remaining variation in reaction probabilities within the binomial logit model. For each 100 m increase in the perpendicular distance from a murrelet to the ship’s course, there was an estimated 58% reduction in the odds of reacting (95% C.I. = 54% - 62% reduction, *P* < 0.001; Table 3). Estimates of disturbance rates were not calculated at distances greater than 850 m perpendicular distance from the ship’s path due to the small sample sizes. If we assumed equal distribution of murrelets across the range of perpendicular distances from the ship, and calculated the cumulative probability of flushing based on the modeled probability at each distance (Fig 5), cruise ships are expected to disturb nearly 100%, 86%, and 68% of all murrelets encountered within a 200 m, 600 m, and 850 m perpendicular distance from the ship, respectively.

**Table 3:** Results from a binary logit model of the probability of a murrelet exhibiting a response (either flushing or diving) to an approaching cruise ship in Glacier Bay National Park, Alaska, 2011–2012.

Variable	Β	Odds Ratio	95% C.I.	*P*-value
Intercept	4.212	67.461	43.623–106.659	< 0.001
Perpendicular distance from path (100 meters)	-0.871	0.419	0.382–0.457	< 0.001
Days since June 1	0.008	1.008	1.0004–1.016	0.037
Location				
Upper Bay (ref)	1	---	---	---
Lower Bay	0.878	2.407	1.780–3.272	< 0.001
AUC statistic = 0.885				

Focal murrelets in the Lower Bay exhibited a greater propensity to react to approaching cruise ships compared to those in the Upper Bay; there was a 141% increase in the odds of reacting at any given distance for murrelets in the Lower Bay compared to murrelets in the Upper Bay (95% C.I. = 78% - 227% increase, *P* < 0.001; Table 3). There was also a slight but significant positive seasonal effect on the probability of murrelets reacting to an approaching cruise ship. On average, the probability of murrelets reacting to a cruise ship tended to be higher at slightly greater perpendicular distances to the ship as the season progressed, providing no evidence of habituation to cruise ship traffic. This resulted in a 0.8% increase in the odds of reacting for each additional day after June 1 (95% C.I. = 0.04% - 1.6% increase, *P* = 0.037; Table 3). Management-relevant covariates, including number of ships in the Bay per day, ship speed, and ship distance to shore, did not explain a significant proportion of the variation in the probability of a murrelet responding to an approaching cruise ship.

### Multistate models

**Fig 6:**
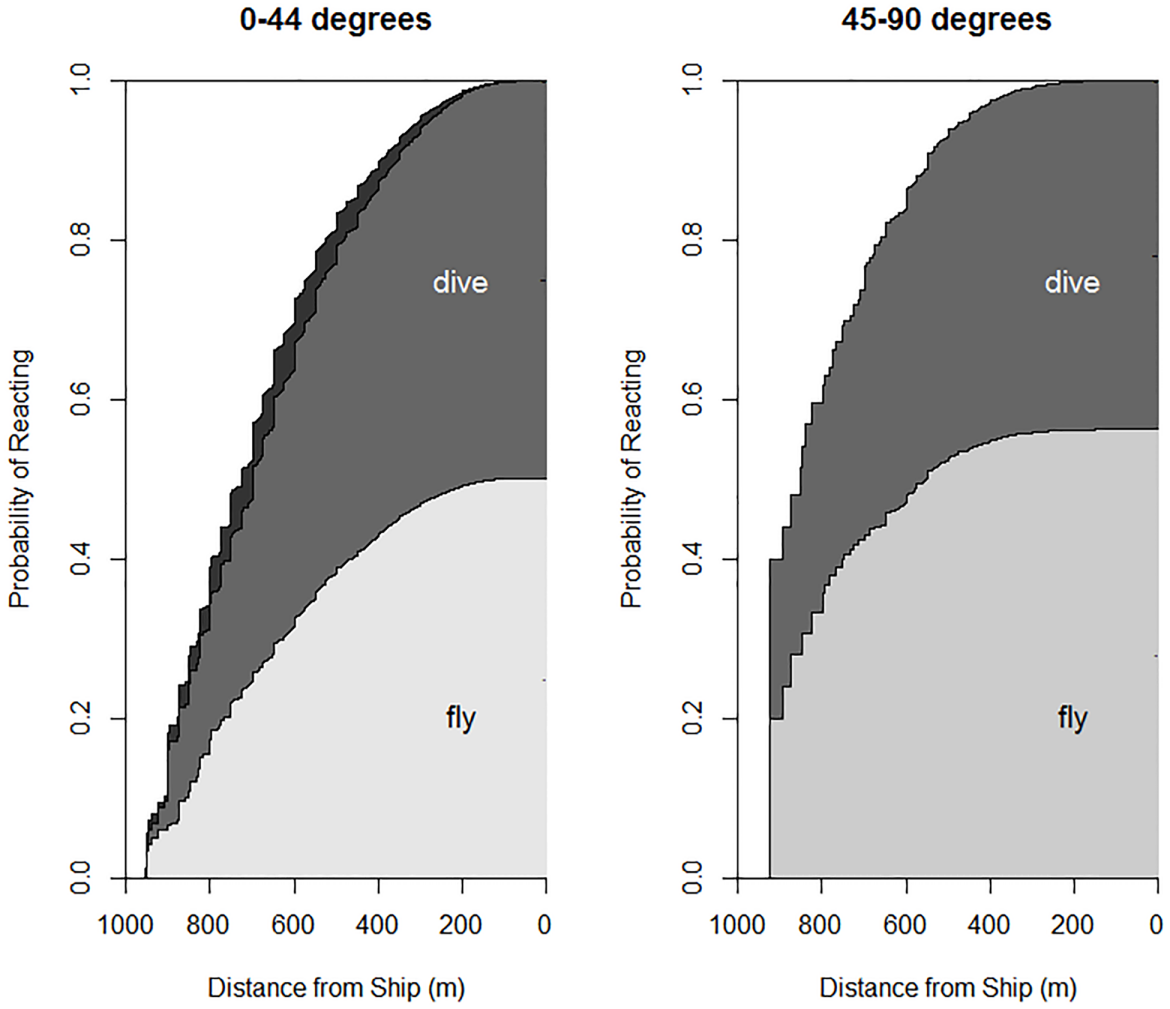
Behavioral transition to flight or diving for murrelets observed from cruise ships at two ship approach angles: 0° - 44° and 45° - 90° from the ship’s course. Note reversed x-axis. Light gray indicates transitions to flight, darker gray indicates transitions to diving, and darkest gray indicates transitions from one bearing bin to the other. Data were collected from cruise ships in Glacier Bay National Park, Alaska, during 2011 and 2012.

When considering absolute distance, rather than perpendicular distance of a murrelet from a cruise ship within the Cox multistate regression model, similar results are evident. Straight-line distance from a murrelet to the cruise ship was a strong predictor of the incidence of both flushing and diving responses, and exhibited a monotonic increase in the observed probability of reaction for murrelets approached by ships both directly and tangentially (Fig 6). On average, murrelets showed a tendency to respond to cruise ships approaching indirectly (45–90 degree approach angle) at greater distances compared with murrelets approached directly (0–44 degree approach angle). Additionally, there appeared to be a slight increase in the proportion of murrelets that responded by flushing, instead of by diving, when approached by the cruise ship indirectly rather than directly.

Similar to the logit models, location in the Park was the only factor that explained a significant proportion of the variation in all four behavioral transition states within the Cox multistate model. Compared to focal murrelets in the Upper Bay, focal murrelets in the Lower Bay exhibited a 91% and a 93% increase in the probability of a diving response when approached by a cruise ship directly and indirectly, respectively, (95% C.I. = 65–121% increase, *P* < 0.001; 49–152% increase, *P* < 0.001; Table 4). Similarly, murrelets in the Lower Bay exhibited a 51% and 34% increase in the probability of a flushing response when approached directly and indirectly, respectively, compared to murrelets in the Upper Bay (95% C.I. = 32–74%, *P* < 0.001; 5–70%, *P* = 0.019; Table 4).

**Table 4:** Hazard ratios explaining the variation in murrelet response type (flush or dive) and distance (m) in a multistate model for transitions from either the 0° - 44° or 45° - 90° cruise ship approach angles and the two response types (flush, dive).

Variable	Description	**0**° **- 44**° **to flight**		**45**° **- 90**° **to flight**	
		Hazard (95% C.I.)	*P*	Hazard (95% C.I.)	*P*
Group	1	1	---	1	---
	≥ 2	1.00 (0.88–1.13)	0.970	1.05 (0.85–1.29)	0.652
Ship Entry	First	1	---	1	---
	Second	0.98 (0.87–1.11)	0.736	0.93 (0.75–1.14)	0.481
Sea State	Light	1	---	1	---
	Moderate	0.96 (0.82–1.12)	0.607	1.07 (0.84–1.41)	0.527
Ship Speed	Knots	1.01 (0.99–1.03)	0.211	0.99 (0.96–1.02)	0.409
Distance to Shore	Km	1.04 (0.95–1.13)	0.440	1.15 (0.98–1.34)	0.089
Stage of Season	Days	1.00 (0.99–1.01)	0.404	1.01 (1.00–1.02)	**<0.001**
Location	Upper Bay	1	---	1	---
	Lower Bay	1.51 (1.31–1.74)	**<0.001**	1.34 (1.05–1.70)	**0.019**
		**0**° **- 44**° **to dive**		**45**° **- 90**° **to dive**	
		Hazard (95% C.I.)	*P*	Hazard (95% C.I.)	*P*
Group	1	1	---	1	---
	2	0.62 (0.55–0.70)	**<0.001**	0.91 (0.73–1.13)	0.385
Ship Entry	First	1	---	1	---
	Second	0.85 (0.75–0.98)	**0.023**	0.99 (0.78–1.24)	0.908
Sea State	Light	1	---	1	---
	Moderate	1.06 (0.90–1.24)	0.487	1.09 (0.81–1.46)	0.568
Ship Speed	Knots	1.01 (0.99–1.02)	0.543	0.98 (0.95–1.01)	0.272
Distance to Shore	Km	0.92 (0.84–1.01)	0.086	0.91 (0.77–1.08)	0.301
Stage of Season	Days	0.99 (0.99–0.99)	**0.002**	1.00 (0.99–1.01)	0.298
Location	Upper Bay	1	---	1	---
	Lower Bay	1.91 (1.65–2.21)	**<0.001**	1.93 (1.49–2.52)	**<0.001**

*P*-values < 0.05 are shown in bold. Data were collected from onboard cruise ships in Glacier Bay National Park, Alaska, during 2011 and 2012.

Season, murrelet group size, and daily number of ships in the Bay each explained a significant amount of the small, but remaining variation in response type, but the effects were not consistent between the two approach angles or the two response types. Advancing season was associated with a decline in the diving response by murrelets when approached directly by a cruise ship, but an increase in the flushing response by murrelets when approached indirectly. With each additional day after June 1, focal murrelets that were approached directly by cruise ships exhibited a 0.5% decline the hazard of diving (95% C.I. = 0.2–0.8%, *P* = 0.002; Table 4), but a 1.1% increase in the hazard of flushing (95% C.I. = 0.4–1.4%, *P* < 0.001; Table 4). Murrelets encountered in groups exhibited a 38% reduction in the incidence of diving when approached directly (95% C.I. = 30–45%, *P* < 0.001; Table 4), although group size was not a significant predictor of murrelet response either when cruise ships approached indirectly or when flushing responses occurred. Similarly, when two cruise ships instead of one entered Glacier Bay during a day, there was a 15% decline in the incidence of a diving response when the cruise ship approached directly (95% C.I. = 2–25%, *P* = 0.023; Table 4), but this effect was not detectable for indirect ship approaches or for flushing responses. No other management-relevant explanatory variables investigated within this study explained a significant proportion of the observed variation in flushing or diving responses to direct or indirect approaches by cruise ships.

### Total number of murrelets disturbed

**Fig 7:**
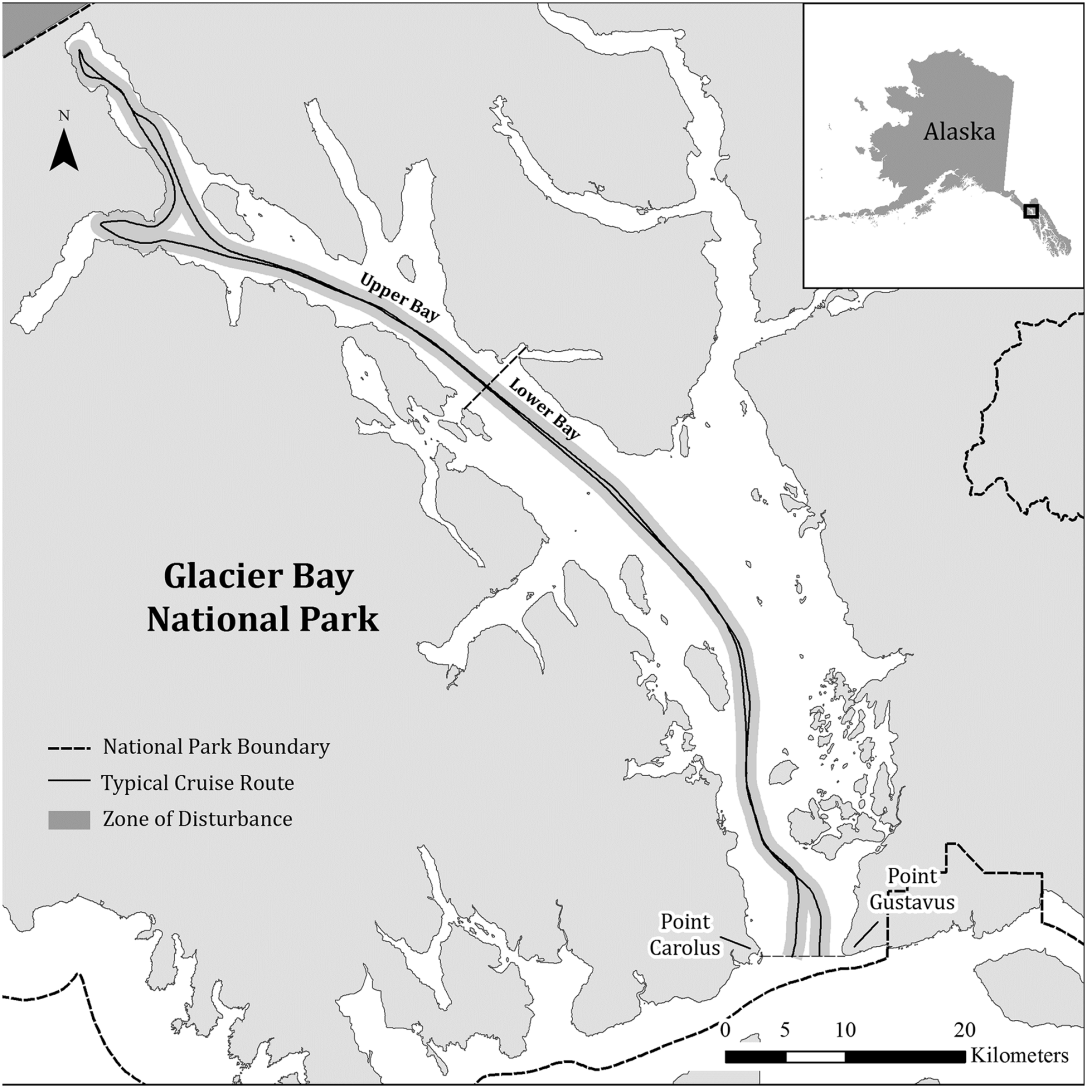
Map of cruise ship disturbance zone in Glacier Bay National Park, Alaska. Gray buffered line indicates the 850 m estimated disturbance buffer around a typical cruise ship track. Solid black line indicates a typical cruise ship route within the Bay. The black bar indicates the dividing line between the Upper Bay and the Lower Bay sectors of the study area. Black dashed line indicates the Park boundary.

Using a typical cruise ship track into and out of the Park, and applying an area out to 850m distance (from the logistic models) on both sides of the track, we estimate that a cruise ship entry into Glacier Bay produces a ‘zone of disturbance’ encompassing approximately 377 km^2^ (Fig 7), assuming the ship follows the same path on exit as it did during entry. Applying a somewhat conservative average probability of flushing (68%) to this area, and an average density of 9.7 Kittlitz’s murrelets and 52.3 marbled murrelets per km^2^ based on surveys from 2009–2015 [28], we estimate that between 15,895 and 31,790 murrelet disturbance events occur each time a cruise ship visits Glacier Bay. The extremes of these estimates reflect the assumption that no additional, or all new murrelets, are disturbed when ships travel back down the Bay and exit the Park. Assuming each disturbance event represents a single murrelet (i.e. a disturbed murrelet does not fly forward of the ship and is disturbed again), the total number of murrelets disturbed represents over 22% of all murrelets in the surveyed area of the Park [28].

## Discussion

Our results clearly demonstrate that a large number of Kittlitz’s and marbled murrelets dive or fly from the water in response to approaching cruise ships in Glacier Bay National Park. Based on over 4,000 murrelet responses recorded during 45 cruises, a modeled average of 68% of all murrelets encountered within 850 m perpendicular distance of either side of the ship dove or flew, creating a 1,700 m ‘zone of disturbance’ centered around the ship’s path. We note that murrelets were likely flushing at greater distances, but detection limits hindered our ability to accurately record responses at distances ≥ 1,000 m.

Based on the results of other studies of alcids responding to vessel traffic [45, 50], including murrelets in Glacier Bay [27], we expected to find that ships elicit disturbance responses. However, the flushing distances recorded in our study were much greater than previously documented for murrelets, and for most other species of waterbirds. For example, in Icy Bay, Alaska, murrelet responses (flying or diving) declined when experimental approach distances were > 150 m [51]. Likewise, in Pacific Rim National Park Reserve, British Columbia, the majority of marbled murrelets did not respond until approaching boats were within 40m [45]. Average flush distances of < 100 m have been demonstrated for foraging waterbirds in Florida [52] and 260 m for foraging guillemots (*Cepphus grylle*) in the Bay of Fundy [50]. In the Baltic and North Seas, median flush distances for flocks of long-tailed ducks (*Clangula hyemali*s) and common eiders (*Somateria mollissima*) were < 300 m, while white-winged and common scoters (*Melanitta fusca* and *M*. *nigra*) flushed at median distances of around 400 and 800 m, respectively [53].

While a number of factors have been shown to account for variation in flushing distances, few studies have considered the importance of vessel size [50, 54], perhaps because the variance in vessel speed and approach angle, in addition to variability in environmental and biological factors, confounds the ability to make such an inference. Yet, the limited number of studies that have examined disturbance to foraging or loafing birds in open marine systems demonstrates a general relationship between vessel size and flushing distances. For example, most of the previously referenced studies utilized vessels 4–10 m in length and found flushing distances of < 200 m. Schwemmer et al. [53] utilized intermediate sized vessel 25–40 m in length and documented intermediate flushing distances (200–800 m). In our study, cruise ships averaged nearly 270 m in length [30] and were typically > 50 m in height. Cruise ships thus produce a much larger visual profile at a particular distance compared to smaller vessels. The results of our survival analysis support this conclusion in that murrelets approached indirectly flushed at greater distances, suggesting that the larger profile of the ships, and the visual stimuli it produced, may have been the mechanism that induced the flushing response.

It is also likely that the responses of conspecifics contributed to the large zone of disturbance created by cruise ships. If murrelets have a greater propensity to flush in response to an approaching cruise ship compared to a smaller vessel at a given distance (e.g., 500 m) it follows that more murrelets will be in flight around cruise ships, providing greater opportunities for other murrelets to flush in response to flushed individuals [55–58]. This may explain, at least in part, the higher incidence of murrelets diving or flying in response to cruise ships in the Lower Bay. Densities of murrelets in the Lower Bay tend to be much higher than in the upper reaches of the Park [28], providing more opportunities for murrelets to respond to flushed conspecifics.

Regardless, the lack of a relationship between flushing distances and management-relevant covariates, such as ship speed, contributes to the uncertainty in management implications. The National Park Service has a mandate to conserve park resources, including the “…dynamics, distributions, habitats, and behaviors of native…plant and animal populations…” in park units [27]. Depending upon assumptions, up to 19% of all murrelets within the surveyed area of Glacier Bay may be disturbed as a result of the transit of a single cruise ship, and perhaps even more on days when the daily maximum of 2 cruise ships enter the Park. What’s more, Kittlitz’s murrelets are thought to be ‘on the edge’ energetically, so the additional resources expended during repeated flights from the water in response to ships may adversely affect individual survival or reproductive success [27].

Yet the NPS also has a mandate to allow visitors to access and enjoy park resources, particularly in Glacier Bay, which was founded, in part, to provide opportunities for people to experience the tidewater glaciers that are “….accessible by ordinary travel…” [59], meaning by marine vessel. While recent NPS policy clarifies that when resource conservation and visitation conflict, resource conservation should prevail [60], it is unclear whether cruise ships are a threat to the conservation of murrelets in the Park, particularly if conservation is measured by abundance. Cruise ships have been visiting Glacier Bay for nearly 2 decades at volumes identical to, or slightly lower than, levels found during our study, yet robust monitoring efforts have not demonstrated any changes in abundance in the Park over the past 8 years [61] and longer [25]. We acknowledge the possibility that the lack of a trend may reflect a population with poor productivity but sustained by immigration of individuals into the Park. However, it is just as possible that the impact of disturbance from cruise ships is not sufficient to depress fitness and, by extension, cause a negative population trend.

Ultimately, continued monitoring efforts, perhaps coupled with studies to better understand the nesting ecology of Kittlitz’s murrelets in Glacier Bay, may help clarify whether cruise ship disturbance rates represent a significant threat to the persistence of murrelet populations in the Park, or if the frequent disturbances represent relatively benign changes in behavior and an acceptable impact from visitation to this marine protected area.

## Supporting information

S1 Appendix Plot of the empirical cumulative distribution of the estimation errors for murrelet distance from ship from two different observers and years in Glacier Bay National Park, Alaska. Black circles; 2011 = a; 2012 = b. Gray dashed vertical lines indicate the 0.025 and 0.975 quantiles (2011 = -144.6 m, 128.6 m; 2012 = -121 m, 120 m). Gray plotted dots indicate a normal distribution based on the mean, standard deviation, and sample size of the error estimates for each observer.
(TIF)
