# Supplementary figures and images for: Disturbance of a rare seabird by ship-based tourism in a marine protected area
Source: PLoS One. 2017 May 10;12(5):e0176176. doi: 10.1371/journal.pone.0176176 (PMC5425178; doi:10.1371/journal.pone.0176176)

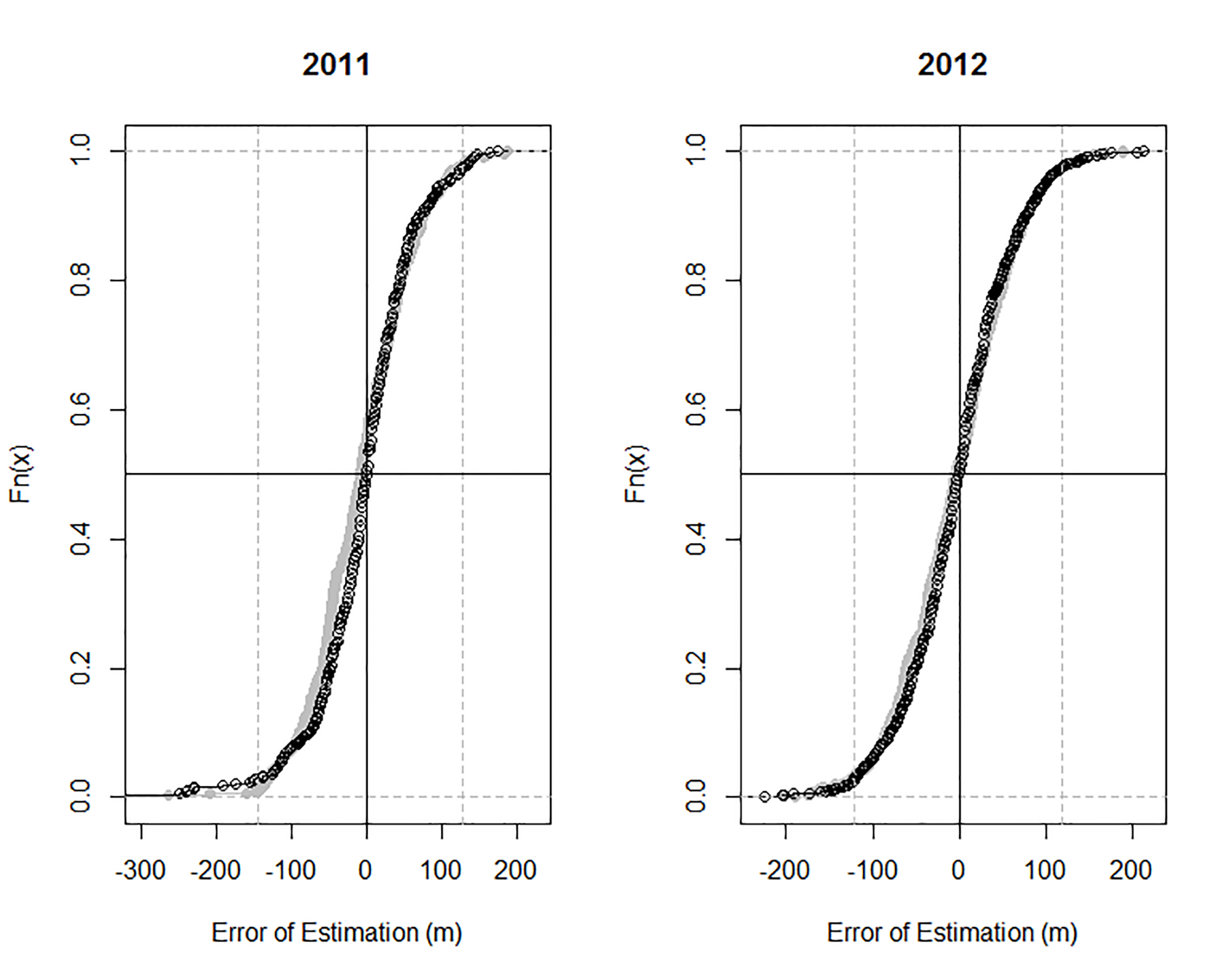

Supplement: S1 Appendix — Black circles; 2011 = a; 2012 = b. Gray dashed vertical lines indicate the 0.025 and 0.975 quantiles (2011 = -144.6 m, 128.6 m; 2012 = -121 m, 120 m). Gray plotted dots indicate a normal distribution based on the mean, standard deviation, and sample size of the error estimates for each observer. (TIF) [file pone.0176176.s001.tif]
